# Reply to: Intergenerational nutrition benefits of India’s national school feeding program: Reality or a bridge too far?

**DOI:** 10.1038/s41467-022-33340-7

**Published:** 2022-10-27

**Authors:** Suman Chakrabarti, Samuel Scott, Harold Alderman, Purnima Menon, Daniel Gilligan

**Affiliations:** 1 University of Washington, Seattle, USA; 2 Poverty Health and Nutrition Division, International Food Policy Research Institute, Washington, DC USA

**Keywords:** Epidemiology, Public health, Developing world, Nutrition

**replying to** H. S. Sachdev et al. *Nature Communications* 10.1038/s41467-022-33338-1 (2022)

We thank Sachdev et al. for engaging with our original article^1^ that explored whether India’s national school feeding program has intergenerational nutrition benefits. Here, we reply to the critiques raised in their Matters Arising (MA) and clarify important aspects of our work related to: (1) the exposure, (2) the pathway from exposure to outcome, (3) the interpretation of the program contribution to future child nutrition, (4) the role of the program in a context of increasing noncommunicable diseases.

## Understanding the exposure definition

The MA states that the same level of Mid-day Meal (MDM) exposure is attributed to all mothers within a state socio-economic status (SES) and birth cohort cell; its variability therein is not considered. We stressed the importance of exploiting variation by SES within states because MDM targets children from poor households. However, NFHS data did not provide information on mother-specific MDM coverage, thus it was impossible to consider variation within the cells used. As mentioned in the original article, our estimates are intent-to-treat (ITT) because the MDM coverage variable measures “potential exposure” to full coverage. The MA claims that highlighting the 0.4 SD increase as the main result (in the abstract) seems an overreach. Here too, we specifically stated that HAZ among children born to mothers with full MDM exposure was greater (+0.40 SD). The 0.4 SD point estimate is an estimate for 100% coverage, whereas the real population effect is smaller due to lower coverage, about 0.053–0.128 SD^1^.

Regarding the argument that MDM exposure is a marker of favorable socio-demographic developmental characteristics, we acknowledged this possibility by stating that the birth cohort model comes at the cost of potential for endogeneity (due to omitted variable bias) because MDM coverage could potentially be associated with changes in living conditions that vary within cohorts defined by state, birth year, and SES strata. This concern was the primary motivation for offering the controlled interrupted time series model (CITS) which does not rely on SES for identification. The MA is justified in pointing out the potential for allocation bias (selection bias where treatment and control groups differ systematically) in quasi-experimental designs, but does not acknowledge the positive inflection in the HAZ trend in intervention states that occurred when MDM was implemented (Figure 5b)^1^. The MA does not provide supportive evidence for socio-demographic factors improving more in intervention states than control states around the time of MDM expansion.

## The pathway from school feeding to improved child linear growth

The MA suggests that mediation analyses should be performed using maternal height and education. Mediation analysis comes with the challenges of accounting for exposure-mediator confounding and mediator-outcome confounding beyond the challenge of accounting for exposure-outcome confounding^2^. There is ample evidence of a strong relationship between education (mediator) and height-for-age z-score (HAZ) (outcome), and this relationship increases as education increases, well beyond mere literacy or primary skills^3^. This evidence plus the well-described link between MDM (exposure) and education (mediator) offer evidence for education being the strongest potential mediating pathway^4,5^. The established mechanism for this pathway is not duly acknowledged in the MA, which emphasizes maternal stature.

The MA quotes our statement in the article that the Indian Human Development Survey (IHDS) households move by one or two SES deciles over 7 years, if they move at all^1^. The contention that intergenerational transmission of favorable socio-demographic characteristics is the primary driver of improved child growth is undermined because there is so little mobility by SES in the short run. It is unclear to us how a substantial change in an outcome such as HAZ could be attributed to a relatively static characteristic. The results of the Delhi study^6^ cited by the MA, which has among the highest SES levels in India, do not directly refute our findings, but also have weak external validity for the relationships we study. While parental height being the primary predictor of attained height is well established^7,8^, the MA mentions neither the evidence from IHDS that MDM is associated with a 1.3 cm height benefit on average nor the combined effects of mediators beyond maternal height such as education, delayed pregnancy, parity and access to health care.

## Interpreting the contribution of MDM to child HAZ

The MA point that child HAZ was lower (overlapping 95% CI) among control mothers prior to the expansion of MDM (Figure 5b) is well taken but this does not violate the core assumptions of the CITS model. The CITS does not require that the lines pre-intervention should overlap, just that the trends be parallel^9^. This assumption was validated by the model.

The MA also states that with more granular adjustments (birth year and state-specific SES fixed effects) or substituting the relatively time-invariant caste and religion for SES (Supplementary Figure 4), MDM was not significantly (P > 0.05) associated with child HAZ. The p-value in the caste/religion regression was 0.06 and articles published in *Nature*^10^ specifically warn against the kind of conclusions the MA attempts to reach. Moreover, we contend that the model that adjusted for the full set of fixed effects, birth year x SES, birth year x State, and State x SES is a case of over control. Since our primary exposure relies on those three features for variation, adding fixed effects for all of their interactions leaves little variation to exploit.

Next, using examples from three states, the MA states that the aggregate data-based outcome interpretation of state-specific changes in nutritional intake attributed to MDM, should therefore be made with abundant caution. Here, they claim that Jharkhand had the MDM and Arunachal Pradesh did not. In our CITS model, Jharkhand and Arunachal Pradesh were categorized as phase three (low coverage before 2004) states. In fact, for the period we analyzed, we did not expect significant MDM-related benefits in Jharkhand. The gains experienced by Arunachal Pradesh do not falsify the benefits of the MDM but rather affirm that there are multiple determinants of child growth^11^, as shown by our decomposition findings that the MDM accounted for 13–32% of the HAZ improvement in India from 2006 to 2016.

## School feeding programs in the context of increasing noncommunicable diseases

Lastly, we share the concern raised in the MA that low-quality cereal-dominated diets may be contributing to the rise of noncommunicable diseases in India. However, this is perhaps a reason for a well-designed adolescent school feeding program rather than a justification for restricting the MDM to only young children. The Comprehensive National Nutrition Survey data from 2016–2018 show that multiple forms of undernutrition are still widespread in India: 24% of adolescents had BMI-for-age < −2 SD while only 1% had BMI-for-age >2 SD, 28% were anemic (40% in females), and deficiencies in iron (22%), folate (37%), B12 (31%) and zinc (32%) were common^12^.

School meals are an opportunity to support health and learning and to reduce hunger. School feeding programs in at least 39 countries target secondary school students and countries with the lowest levels of secondary school feeding programs also have the highest levels of early marriage and pregnancy^13,14^. India’s MDM program should aim to reach all young people throughout their full course of development and to improve meal quality and diet diversity^15^.
